# Enhancement of cellulolytic enzyme production from intrageneric protoplast fusion of *Aspergillus* species and evaluating the hydrolysate scavenging activity

**DOI:** 10.1186/s12934-024-02343-y

**Published:** 2024-03-02

**Authors:** Doaa A. Goda, Huda M. Shakam, Mai E. Metwally, Hager A. Abdelrasoul, Mohamed M. Yacout

**Affiliations:** 1 Bioprocess Development Department, Genetic Engineering and Biotechnology Research Institute (GEBRI), City of Scientific Research and Technological Applications (SRTA-City), Universities and Research Institutes Zone, P.O. 21934, New Borg El-Arab City, Alexandria, Egypt; 2 Genetics Department, Faculty of Agriculture (El-Shatby), Alexandria, Egypt

**Keywords:** Cellulase, *Aspergillus*, Protoplast fusion, Optimization

## Abstract

**Background:**

Lignocellulosic biomass provides a great starting point for the production of energy, chemicals, and fuels. The major component of lignocellulosic biomass is cellulose, the employment of highly effective enzymatic cocktails, which can be produced by a variety of microorganisms including species of the genus *Aspergillus*, is necessary for its utilization in a more productive manner. In this regard, molecular biology techniques should be utilized to promote the economics of enzyme production, whereas strategies like protoplast fusion could be employed to improve the efficacy of the hydrolytic process.

**Results:**

The current study focuses on cellulase production in *Aspergillus* species using intrageneric protoplast fusion, statistical optimization of growth parameters, and determination of antioxidant activity of fermentation hydrolysate. Protoplast fusion was conducted between *A. flavus* X *A. terreus* (PFFT), *A. nidulans* X *A. tamarii* (PFNT) and *A. oryzae* X *A. tubingensis* (PFOT), and the resultant fusant PFNT revealed higher activity level compared with the other fusants. Thus, this study aimed to optimize lignocellulosic wastes-based medium for cellulase production by *Aspergillus spp.* fusant (PFNT) and studying the antioxidant effect of fermentation hydrolysate. The experimental strategy Plackett-Burman (PBD) was used to assess how culture conditions affected cellulase output, the best level of the three major variables namely, SCB, pH, and incubation temperature were then determined using Box-Behnken design (BBD). Consequently, by utilizing an optimized medium instead of a basal medium, cellulase activity increased from 3.11 U/ml to 7.689 U/ml CMCase. The following medium composition was thought to be ideal based on this optimization: sugarcane bagasse (SCB), 6.82 gm; wheat bran (WB), 4; Moisture, 80%; pH, 4; inoculum size, (3 × 10^6^ spores/ml); and incubation Temp. 31.8 °C for 4 days and the fermentation hydrolysate has 28.13% scavenging activities.

**Conclusion:**

The results obtained in this study demonstrated the significant activity of the selected fusant and the higher sugar yield from cellulose hydrolysis over its parental strains, suggesting the possibility of enhancing cellulase activity by protoplast fusion using an experimental strategy and the fermentation hydrolysate showed antioxidant activity.

## Background

The use of plant biomass as a renewable raw material, particularly as an unending supply of low-cost carbohydrates, has been gaining traction. It is a potential substrate to produce a range of valuable products [1]. The phrase “lignocellulosic biomass” refers to plant biomass, which includes forestry, agricultural, agro-industrial, and food wastes [2]. Because lignocellulose from various agricultural residues varies in composition, it is primarily made up of lignin, cellulose, hemicelluloses, and pectin in plant cell walls, the process of hydrolyzing lignocellulose to produce simple monomeric sugars requires complex enzyme mix [3]. Effective cellulase manufacturing techniques are essential to meet the growing need for affordable and sustainable enzymatic processes, as cellulases are significant industrial enzymes utilized in a range of sectors [4].

Numerous bacteria and filamentous fungi are known to release enzymes that hydrolyze lignocelluloses [4]. Fungi are essential sources of hydrolytic enzymes in industry. Lignocellulolytic fungi are extensively spread throughout the fungal kingdom, and many of them are filamentous fungi because they are simple to grow and produce a lot of extracellular enzymes [5]. The genus *Aspergillus* has been used successfully as a host to produce recombinant proteins of commercial enzyme cocktails [6]. According to the development of genetic transformation systems, scientists may now convert foreign DNA into filamentous fungus and produce the desired strains for usage in industry. A variety of techniques are used to develop industrially important microorganisms, protoplast fusion is the most straightforward and widely applied method which permits the recombination of genomes from many parental strains [7, 8]. It can be seen as one of the recombinant DNA technologies that offer the ability to enhance the dosage and expression of genes from potent promoters, eliminate undesirable genes from the fungal genome, modify metabolic pathways, and create fungal strains that can generate heterologous proteins [9].

Agro-industrial by-products are becoming more and more sought-after as a source of antioxidant compounds worldwide. As a result, efforts to extract antioxidant compounds using alternative green methods have been developed. Traditional extraction methods that use organic solvents hurt the environment. Enzymes are used to release bioactive substances through antioxidant activity [10].

Therefore, the current study aimed to generate improved recombinant strains through intrageneric protoplast fusion between local *Aspergillus spp.* isolates which is a promising way to increase cellulase output, those can utilize sugarcane bagasse and wheat bran as agriculture residues under solid-state fermentation (SSF) to overproduce cellulases and evaluate their activities, refine the fermentation medium to achieve the highest level of productivity, and determining antioxidant effect of the fermentation hydrolysate.

## Methods

### Fungal strains

The identified fungal isolates *Aspergillus flavus* RCMB 002F003(3), *A. nidulans* RCMB 002F021(1), *A. oryzae* RCMB 002F018(1), *A. tamarii* RCMB 002F005(1), *A. terreus* RCMB 002F007(1) and *A. tubingensis* RCMB 002F032(1) were obtained from the Collection of Microorganisms from the Regional Center for Mycology and Biotechnology, Al-Azhar university, Cairo, Egypt. These strains were selected according to their cellulase production as described in Table 1. All strains were maintained on Potato Dextrose Agar media (PDA) plates and grown for 7 days at 30 °C and then stored at 4 °C.

**Table 1:** Enzyme activity of *Aspergillus* parents and fusants isolates using solid substrate SCB and WB combined in a ratio of 1:1 as carbon source for cellulose production

Culture	Cellulase (CMCase) activity (U/ml)	
	2nd day	4th day
Flv	1.78 ^c^	1.29 ^c^
Nid	4.53 ^a^	2.04 ^b^
Ory	1.54 ^c,d^	1.19 ^c^
Tam	1.98 ^c^	2.37 ^a,b^
Ter	1.9 ^c^	0.98 ^c^
Tub	0.87 ^e^	1.21 ^c^
PFFT	1.17 ^d,e^	0.87 ^c^
PFNT	3.11 ^b^	2.64 ^a^
PFOT	1.8 ^c^	0.93 ^c^

*Note* The same letters within a column indicate that the values are not significantly different at the *p* = 0.05 level

### Protoplast preparation and fusion

Protoplasts were produced via actively developing *Aspergillus* mycelium employing enzyme lysis following the method of Kaur et al. [11] with a few alterations. One ml of a homogeneous spore suspension (10^5^ spores/ml) of *Aspergillus* spp. was added to 25 ml of broth potato dextrose medium enhanced with 1.5% yeast extract. The flask was then kept on shaker at 30 °C and 150 rpm for sixteen hours, then 1 ml from mycelial content was centrifuged for ten minutes at 10,000 rpm. After discarding the supernatants, an osmotic stabilizer was used three times to wash the mixture. For the release of protoplasts, add 8 mg/mL of lysing enzyme (from *T. harzianum*, Sigma-Aldrich) produced in 0.2 M phosphate buffer pH 6.0 with 0.6 M KCl for three hours at 30 °C and 100 rpm., the cultures filtered via four sterilized sections of cheesecloth, the supernatant with protoplasts were carefully agitated for two minutes at 2,000 rpm. Next, a concealed quantity of osmotic stabilizer was used to resuspend the protoplasts. For two minutes, one milliliter of 10^6^ milliliter^− 1^ protoplasts from each parental strain was combined and centrifuged at 2,000 rpm. Following the formation of the protoplast pellet, it was again suspended in 2 milliliters of freshly made polyethylene glycol (30%, w/v; PEG 4000 MW), which was made in 0.05 M NaOH-glycine buffer (pH 7.5) with 0.05 M CaCl2. After giving the mixture a gentle shake, it was left at room temperature for 45 min. Following dilution, 0.1 ml of the fused protoplast sample was combined with 4 ml of soft agar regeneration medium. After that, the mixture was spread out across plates that included hard agar regeneration medium with sucrose added as an osmotic stabilizer. After that, plates were incubated at 30 °C to promote colony growth and protoplast production. The new resultant fusants were namely *A. flavus* X *A. terreus* (PFFT), *A. nidulans* X *A. tamarii* (PFNT), and *A. oryzae* X *A. tubingensis* (PFOT).

### Enzyme production in solid-state fermentation (SSF) at flask scale

SSF experiments were carried out according to the method of [12] with minor modifications, for inoculum preparation (spores’ suspension) the parental strains and the fusants were grown separately in a PDA medium and incubated for 5–7 days at 30 °C, then the SSF medium prepared as the following :2.5 g of raw sugarcane bagasse (SCB) and 2.5 g of raw wheat bran (WB) were combined in a ratio of 1:1 and supplemented with nutrient solution (w/v%: (NH_4_)_2_SO_4_, 0.35; KH2PO4, 0.3; MgSO4.7H2O, 0.05; and CaCl_2_, 0.05) for a 65% moistening level, then autoclaved. Following sterilization, each flask of each parental strain and fusants was inoculated with 1 ml of (2 × 10^6^ spores’ suspension) and incubated at 30 °C in duplicate.

### Evaluation of cellulase activity

The enzyme extract was generated by mixing fermented ingredients with 50 ml of sterile distilled water, homogenizing then incubated it at 150 rpm for 60 min. Later, the components were filtered through cheesecloth and centrifuged twice for 10 min at 10,000 rpm. The resulting supernatants have been separated and used as crude enzymatic lysates for quantitative evaluation. After that the cellulase activity was measured by the method of [13] using 0.5% Carboxymethyl Cellulose (CMC) as a substrate at pH 4.8 (0.05 M Citrate buffer) and 50 °C. At 540 nm, the soluble fraction’s absorbance was calculated in relation to the blank. With glucose, a standard curve was produced. Under the experimental conditions, one unit of cellulase activity was defined as the amount of enzyme that released one µmol of glucose per minute.

### SSF Statistical optimization for cellulase production

The parameters of cellulase production under SSF were statistically optimized with the Plackett-Burman Design (PBD) for factors screening, and then the most important parameters that affect the enzyme production were submitted to Box-Behnken Design (BBD) for enhanced activity according to [14].

The PB experimental design was used to assess the relative importance of seven variables (SCB, WB, moisture %, pH, T (◦C), incubation time, and inoculum size) affecting the PFNT fusant ability to produce cellulase in SSF, across a set of 12 trials. PBD used the first-order model: the response *(Y) =* the model intercept *(β0) + Σ* [the variable estimate (βi) * the variable *(xi)*]. The standard regression *p-value* evaluated the significance of the variables. The investigated factors and the assessed values of each component are shown in Table 2. Each component was compared at high (+ 1) and low (-1) levels (Table 2).

**Table 2:** Funding this research factors influencing cellulase production by the fusant (PFNT) using solid substrate SCB and WB combined in a ratio of 1:1 as carbon source

Trials	Variables							Cellulase (U/ml)		
	X1	X2	X3	X4	X5	X6	X7	Experimental	Predicted	Residual
**1**	-1	-1	-1	1	-1	-1	1	1.592715	1.807955	-1.09599
**2**	1	1	1	-1	-1	-1	1	3.710501	3.433152	1.412246
**3**	-1	1	-1	1	1	1	-1	1.367739	1.090389	1.412246
**4**	-1	1	1	1	-1	-1	-1	1.92526	2.032159	-0.54432
**5**	1	1	1	1	1	1	1	1.977767	2.166343	-0.96022
**6**	-1	-1	1	-1	-1	1	-1	2.669111	2.535549	0.680089
**7**	-1	-1	1	-1	1	1	1	1.592715	1.726277	-0.68009
**8**	1	-1	-1	1	-1	1	1	2.966651	2.751411	1.095987
**9**	1	1	-1	-1	-1	1	-1	2.996641	3.300654	-1.54802
**10**	-1	1	-1	-1	1	-1	1	1.592715	1.547927	0.228061
**11**	1	-1	-1	-1	1	-1	-1	2.327815	2.34594	-0.09229
**12**	1	-1	1	1	1	-1	-1	2.039026	2.02090	0.092292
**Variables**		**Code**	**Coded level and actual level**							
			**-1**	**1**						
**SCB (gm)**		X1	2	4						
**WB (gm)**		X2	2	4						
**Moisture (%)**		X3	60	80						
**pH (value)**		X4	4	6						
**Temp. (°C)**		X5	30	37						
**Time (days)**		X6	4	8						
**Inoculum size (sp/ml)**		X7	1 × 10^6^	3 × 10^6^						

Table 3, the BB experimental design for Response surface methodology (RSM) was exploited to get the optimal values of the most significant three variables, across a set of 14 trials with two central points, three levels (high + 1, medium 0, and low − 1) for the chosen variables and the measured response [15]. Performing the statistically designed trials, estimating the coefficients of the structured mathematical model, projecting the response, and evaluating the model’s suitability were the three primary processes in this optimization process [16]. Utilizing each variable’s coefficient data, the developed model was applied [17]. BBD used the second-order polynomial structured model according to [14].

**Table 3:** BB designed matrix for the selected 3-variables (X1 _Sugarcane bagasse_, X2 _pH_ and X3 _Temperature_) influencing cellulase production by the fusant (PFNT) using solid substrates SCB and WB.

	Variables			Cellulase (U/ml)		
Trial	X1	X2	X3	Experimental	Predicted	Residual
**1**	**-1**	**-1**	**0**	3.692999	4.617845	-0.92485
**2**	**1**	**-1**	**0**	7.269158	7.323616	-0.05446
**3**	**-1**	**1**	**0**	7.428098	7.373640	0.05445
**4**	**1**	**1**	**0**	8.343898	7.419052	0.92484
**5**	**-1**	**0**	**-1**	5.489357	5.500473	-0.01112
**6**	**1**	**0**	**-1**	4.404210	5.285714	-0.88150
**7**	**-1**	**0**	**1**	5.307947	4.426443	0.88150
**8**	**1**	**0**	**1**	7.403500	7.392384	0.01111
**9**	**0**	**-1**	**-1**	4.113056	3.177093	0.93596
**10**	**0**	**1**	**-1**	4.655629	4.698971	-0.04334
**11**	**0**	**-1**	**1**	3.833018	3.789676	0.04334
**12**	**0**	**1**	**1**	4.183065	5.119028	-0.93596
**13**	**0**	**0**	**0**	5.863292	6.012062	-0.14877
**14**	**0**	**0**	**0**	6.160833	6.012062	0.14877
**Variables**	**code**	**Coded level and actual level**				
		**-1**	**0**	**1**		
**SCB**	X1	3	5	7		
**pH**	X2	3	4	5		
**Temp.ºC**	X3	25	30	35		

### The statistical evaluation of data

The enzyme activity variation for the fusants and the parental strains was analyzed by using the data analysis tools in the Microsoft Excel program. For the fusant PFNT-related enzyme activity data, the JMP program was used to estimate the *t* values, *p* values, confidence levels, and the *p* values as a percentage to assess the activity’s optimal value. A three-dimensional graph created by STATISTICA 8.0 software was used to illustrate the simultaneous effects of the three most significant independent factors on each response [14].

### Antioxidant activity (DPPH-free radical-scavenging assay)

The scavenging activity of the fermentation hydrolysate after the optimization process was assessed through free radical scavenging activity (RSA) using 1,1-diphenyl-2-picrylhydrazyl (DPPH), and the RSA efficiency was compared to ascorbic acid using a minor modified method described previously [18]. Briefly, 400 µL of hydrolysate solutions (The enzyme hydrolysate produced by fusant PFNT after the optimization process was extracted as mentioned before) were mixed with 1 mL of 100 µM DPPH. To prepare the negative control test, 400 µL of absolute methanol was added to 1 mL of DPPH. The reactions were kept in the dark for 30 min at room temperature, and the discoloration of DPPH was seen at a wavelength of 517 nm.

The RSA scavenging efficiency was calculated according to:

### RSA % = [$\frac{ \text{C}\text{o}\text{n}\text{t}\text{r}\text{o}\text{l} \text{A}\text{b}\text{s}\text{o}\text{r}\text{b}\text{a}\text{n}\text{c}\text{e}-\text{S}\text{a}\text{m}\text{p}\text{l}\text{e} \text{A}\text{b}\text{s}\text{o}\text{r}\text{b}\text{a}\text{n}\text{c}\text{e}}{ \text{C}\text{o}\text{n}\text{t}\text{r}\text{o}\text{l} \text{A}\text{b}\text{s}\text{o}\text{r}\text{b}\text{a}\text{n}\text{c}\text{e}}$ ]*100

## Results

### Selection and screening of the fusants

**Fig. 1:**
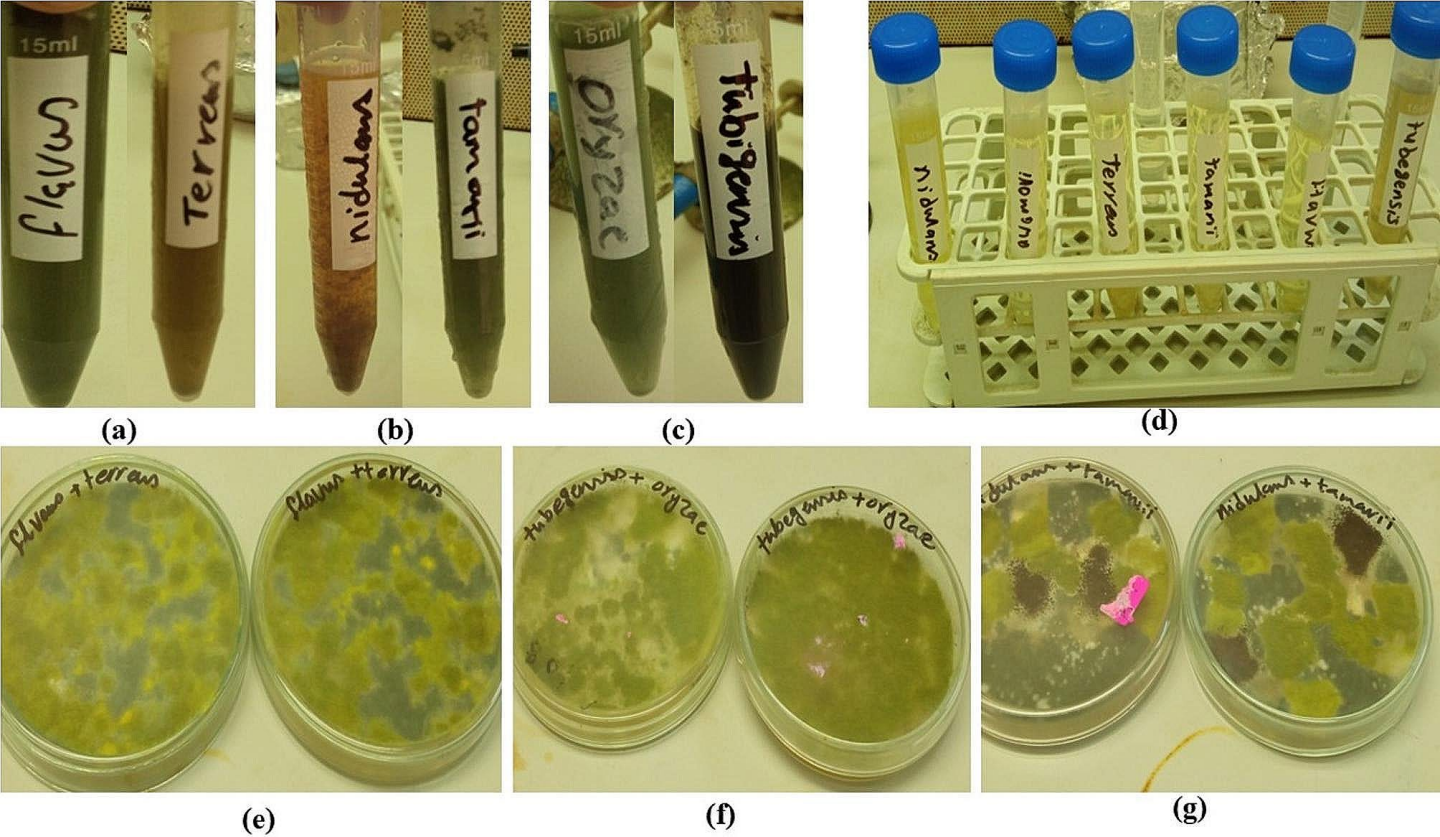
Protoplast Preparation and Fusion, where **(a)** spores’ suspensions of *A. flavus* and *A. terreus***(b)** spores’ suspensions of *A. nidulans* and *A. tamarii*, **(c)** spores’ suspensions of *A. oryzae* and *A. tubingensis*, **(d)** protoplasts released in presence of Lysing Enzyme, **(e)** Heterokaryons exhibiting intermediate spore coloration *A. flavus* X *A. terreus*, **(f)** Heterokaryons exhibiting intermediate spore coloration *A. nidulans* X *A. tamari*, and **(g)** Heterokaryons exhibiting intermediate spore coloration *A. oryzae* X *A. tubingensis*

The current study reports interspecific protoplast fusion between each of the two tested cellulolytic strains (Fig. 1a,b,c). The protoplasts were released in the presence of lysing enzymes (Fig. 1d) and fused in the presence of 30% PEG then plated on a regeneration medium and incubated at 30 °C. Heterokaryons, exhibiting intermediate spore coloration (Fig. 1e,f,g), were further evaluated for extracellular production of cellulase under solid substrate culture at 30 °C.

**Fig. 2:**
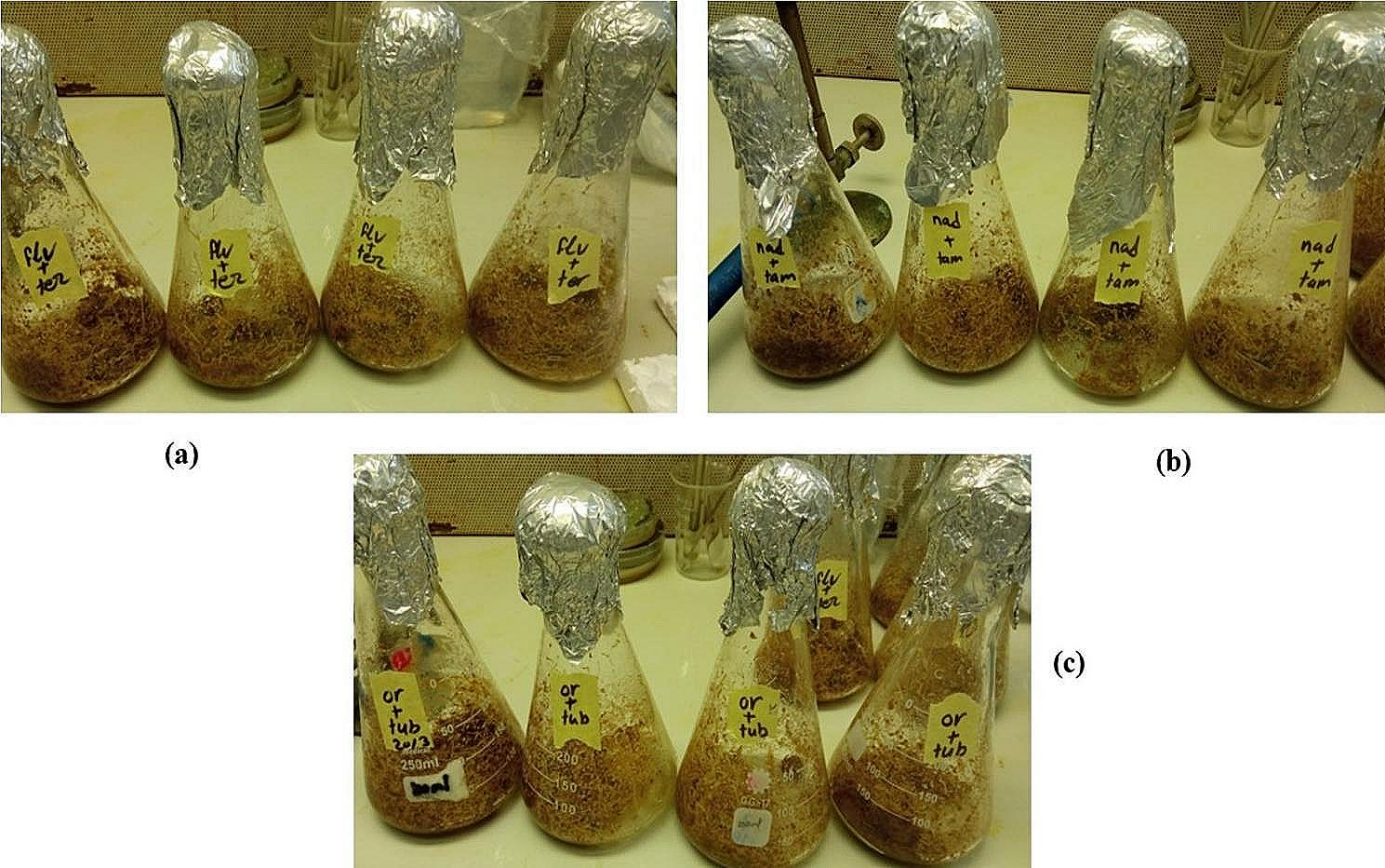
Growth of **(a)** PFFT, **(b)** PFNT and **(c)** PFOT on SSF production medium of carboxymethyl cellulase

The results in Table 1 show the production of endoglucanase (carboxymethyl cellulase) by the parental strains *A. flavus* (Flv), *A. nidulans* (Nid), *A. oryzae* (Ory), *A. tamarii* (Tam), *A. terreus* (Ter), and *A. tubingensis* (Tub) and the fusants PFFT, PFNT and PFOT on solid-state production medium (Fig. 2) comprising a combination of SCB and WB as carbon source. On the second incubation day, the enzyme activities were higher for most of the strains and decreased by the fourth day. On the contrary for Tam and Tub strains, activity was increased by the fourth day of incubation. Nid and the fusant PFNT showed higher enzyme production efficiency 4.53 and 3.11 U/ml, respectively compared with the other parental strains and fusants. To figure out the ideal circumstances needed for enzyme activity in crude extract, fusant PFNT was selected and examined with different parameters.

**Fig. 3:**
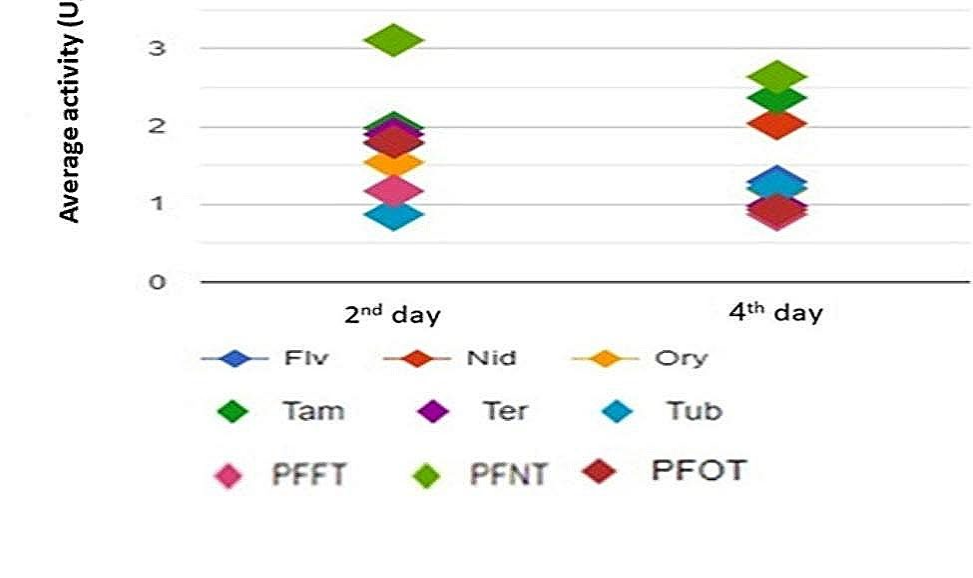
The Results of means comparisons of enzyme activity between *Aspergillus* parental strains and fusants at the second and forth days of incubation under Solid-State Fermentation

According to the ANOVA fixed-test using F distribution (Fig. 3), Factor A (difference between the enzyme activity at the second and fourth incubation day for each sample), since the *p-value* > 0.05, which is the difference between the sample averages of all groups is not big enough to be statistically significant. While Factor B (difference between the enzyme activity for each sample), since the *p-value* < 0.05, which is the sample difference between the averages of some groups is big enough to be statistically significant.

### Multifactorial experiment with statistics for cellulase synthesis

To assess the relative importance of culture factors influencing cellulase production by *Aspergillus* fusant strains, the PBD design was used. According to the regression analysis in Table 4, the correlation coefficient of the seven factors, including SCB, WB, moisture, inoculum size, and cultivation period, had a favorable impact on cellulase activity. However, pH and culture temperature were discovered to have a deleterious impact. Based on the formula confidence level (%) = (1- *pvalue*) ×100, the linear multiple regression analysis approach was used to analyze the 7 variables. Aside from that, the major effect was essentially determined as the difference between the average values of every variable obtained at two levels: one at a high level (+ 1) and a second at a low level (-1) (Table 2).

**Table 4:** Statistical analysis of PBD showing coefficient values, main effect, t and p values, and confidence level % for each variable affecting cellulase production using solid substrates SCB and WB combined in a ratio of 1:1

Term	Coefficients	Main effect	Standard Error	t Stat	P-value	Confidence level (%)
**Intercept**	2.229888		0.094014	23.71868	0.0000187	96.1301
**Sugarcane bagasse**	0.439845	0.879691	0.094014	4.678510	0.009458	24.84297
**Wheat bran**	0.031882	0.063765	0.094014	0.339127	0.751570	60.34304
**Moisture**	0.089175	0.178351	0.094014	0.948532	0.396570	94.46109
**pH**	-0.251695	-0.50339	0.094014	-2.677210	0.055389	98.83021
**Temp.**	-0.413591	-0.82718	0.094014	-4.399260	0.011698	24.84297
**Time**	0.031882	0.063765	0.094014	0.339127	0.751570	7.131335
**Inoculum size**	0.008956	0.017912	0.094014	0.095264	0.928687	24.84297
**ANOVA analysis**	***df***	***SS***	***MS***	***F***	***Significance F***	
**Regression**	7	5.255258	0.750751	7.078311	0.038698969	
**Residual**	4	0.424254	0.106064			
**Total**	11	5.679513				
**Multiple R**	0.96192563					
**R Square**	0.92530091					
**Adjusted R Square**	0.79457751					

The correlation between the variables was examined at a 90% or greater level of confidence using the *p-value* from the ANOVA analysis for each response. At a confidence level of 96.13%, the analysis of variance using the ANOVA test yields *p* = 0.0387, indicating that there is a statistically significant relation between the variables. The correlation R-squared value, the fitted model accounts for 92.53% of the variability in the measured response, which is cellulase activity. The following is a possible presentation of the polynomial model describing the correlation between the cellulase activity and the seven factors.: *Y = 2.22988805 + 0.43984547 × *_*1*_ *+ 0.03188269 × *_*2*_ *+ 0.08917534 × *_*3*_ *− 0.251695 × *_*4*_ *− 0.4135919 × *_*5*_ *+ 0.03188269 × *_*6 +*_*0.00895617 × *_*7*_.

**Fig. 4:**
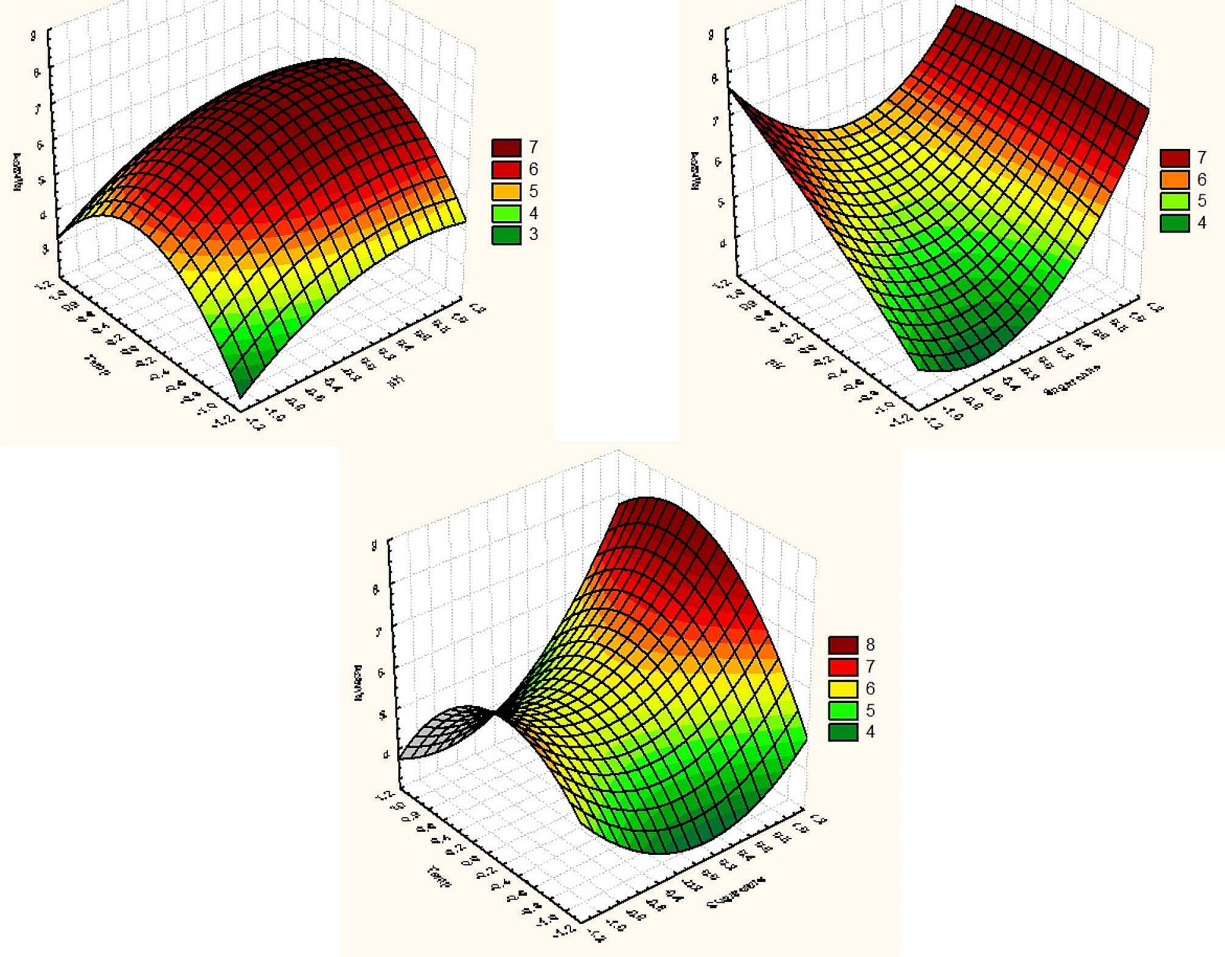
Three-dimensional surface and contour plots showing the relationships between the tested variables and the cellulase produced by the fusant (PFNT).

The three factors that were chosen for the statistical analysis were the amount of SCB, the initial pH, and the temperature since they have a considerable impact on the production of cellulase and have a confidence level of more than 93%. These findings suggest a medium with the following conditions: WB, 4 gm; Moisture, 80%; inoculum size, (3 × 10^6^ spores/ml); and incubation time, 4 days, with enzyme activity 3.710501 U/ml, was used as the foundational medium for subsequent design. The major independent factors (X1, SCB; X2, pH; and X3, Temp.) were further investigated, each at three levels, to determine the optimal response region for cellulase production in terms of activity (U/ml) (Table 3). The three variables were examined using a fourteen-trial linear multiple regression analysis method, and as previously established percentage confidence levels (%) were computed. The value of the determination coefficient *R*^2^ = 0.834 for cellulase activity (Table 5), being a measure of fit of the model, suggests that around 16.6% of the overall fluctuations are not explained by cellulase activity. Surface plots, which display the experimental results, demonstrate that higher levels of cellulase activity were achieved with moderate pH and temperature and higher quantities of SCB. (Fig. 4). A non-linear optimization approach was used to fit the experimental data with a second-order polynomial function in order to predict the variable’s ideal location within the constraints of the experiment.

**Table 5:** Statistical analysis of BB design showing *coefficients*, *t –*and *p-values* for significant variables (X1 _Sugarcane bagasse_, X2 _pH_ and X3 _Temperature_) affecting on cellulase production by the fusant (PFNT) using solid substrates SCB and WB.

Term	Coefficients	Standard Error	t Stat	P-value	Upper 95.0%
**Intercept**	6.012062	0.796165	7.55127	0.00164	8.22257
**x1**_**SCB**_	0.687796	0.398083	1.72777	0.15909	1.79305
**x2**_**pH**_	0.712807	0.398083	1.79060	0.14784	1.81806
**x3**_**Temp**_	0.25816	0.398083	0.64850	0.55200	1.36341
**x1**_**SCB**_ *x2_**pH**_	-0.66509	0.562974	-1.18139	0.30289	0.89797
**x2**_**pH**_ *x3_**Temp**_	0.795175	0.562974	1.41245	0.23067	2.35824
**x1**_**SCB**_ *x3_**Temp**_	-0.04813	0.562974	-0.08550	0.93597	1.51493
**x1**_**SCB**_ *x1_**SCB**_	1.063269	0.629424	1.68927	0.16643	2.81082
**x2**_**pH**_ *x2_**pH**_	-0.39179	0.629424	-0.62246	0.56736	1.35576
**x3**_**Temp**_ *x3_**Temp**_	-1.42408	0.629424	-2.26251	0.08644	0.32348
**ANOVA**	***df***	***SS***	***MS***	***F***	**Prob > F**
**Model**	9	25.61478	2.846087	2.244976	0.22652
**Error**	4	5.071034	1.267758		
**C. Total**	13	30.68582			
**RSquare**	0.834				
**RSquare Adj**	0.46				

*Y = 6.012062 + 0.687795 × *_*1*_ *+ 0.712807 × *_*2*_ *+ 0.258159 × *_*3*_ − 0*.66508 × *_*1*_* × *_*2*_ *+ 0.795175 × *_*2*_* × *_*3*_*-0.0481317 × *_*1*_* × *_*3*_ *+ 1.063268 (X*_*1*_*)*^*2*^*- -0.39179 (X*_*2*_*)*^*2*^*− 1.424077 (X*_*3*_*)*^*2*^.

**Fig. 5:**
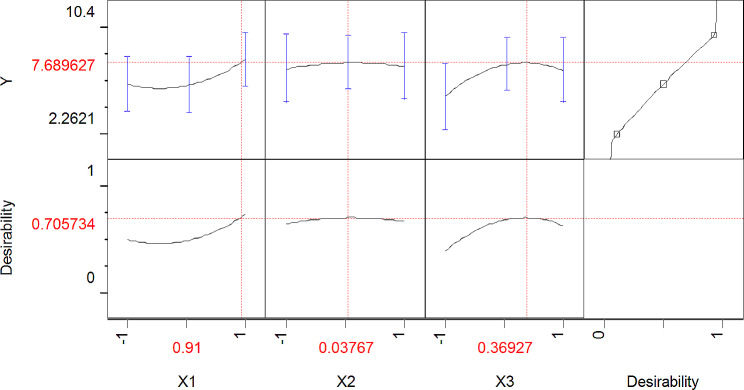
JMP Desirability prediction profle showing the predicted optimal coded levels (0.91, 0.037 and 0.0369) of studied three variables Sugarcane bagasse, pH and Temp, respectively for cellulase production by the fusant (PFNT).

The JMP Desirability prediction profile displays the expected ideal coded levels (0.91, 0.037, and 0.0369) for the three variables under study—sugarcane, pH, and temperature for the fusant’s ability to produce cellulase (PFNT) as shown Fig. 5, where the polynomial model’s maximum point revealed the following values for the three variables under study as the ideal levels: SCB, 6.82 gm; pH, 4; and incubation temperature, 31.8 °C; with predicted estimated enzyme activity equal to 7.689 U/ml. Eventually, An investigation for verification was conducted under anticipated ideal conditions while keeping an eye on the enzyme activity in the ideal medium to assess the quadratic polynomial accuracy. The percentage accuracy was determined using the following formula to demonstrate the model accuracy: Model accuracy is equal to [*Y*_Experiment_/ *Y*_Calculated_] / 100.The results of the verification test indicate that the *Y* value is 8.72 U/ml. The accuracy of the computed model was 113.4%. In this work, a statistical technique that combined PB and BB designs for determining the best concentrations of the statistically relevant components proved to be efficient and accurate. Consequently, it is anticipated that the following medium composition will be close to the ideal: SCB, 6.82 gm; WB, 4 gm; Moisture, 80%; pH, 4; inoculum size, (3 × 10^6^ spores/ml); and incubation Temperature 31.8 °C for 4 days.

**Antioxidant activity of*Aspergillus spp.*fusant (PFNT) fermentation hydrolysate**.

**Fig. 6:**
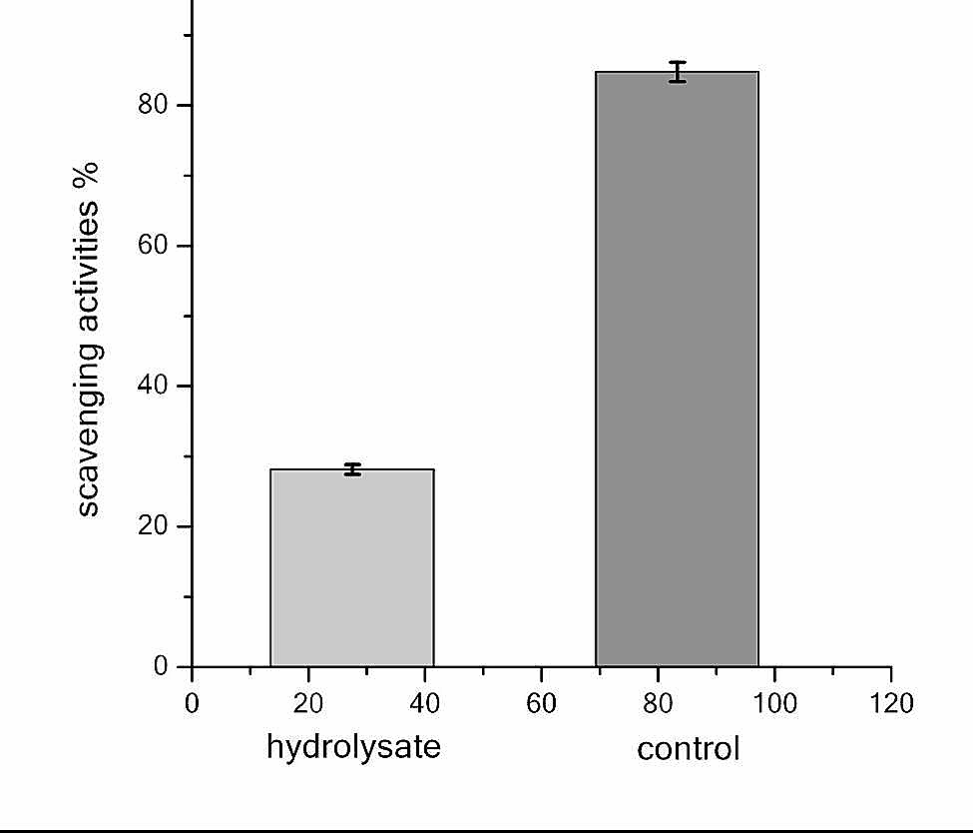
Free radical scavenging efficiency for the fermentation hydrolysate (left) and ascorbic acid as positive control (right)

The antioxidant activity was assessed using the DPPH test of fusant (PFNT) fermentation hydrolysate, it showed a positive response in RSA% where it was 27.13% scavenging activities (Fig. 6).

## Discussion

The natural ability of filamentous fungi to produce huge amounts of industrial hydrolytic enzymes on a big scale has led to substantial research on heterologous protein expression in these organisms. In a typical protein expression, a fungal cell factory expresses a chosen gene that encodes the target enzyme [19, 20]. Cellulase production using cost-effective raw materials in SSF by fungal systems and the ability of *Aspergillus* species to hydrolyze a wide range of carbohydrates to simple sugars is well-known [21]. According to our obtained results, all *Aspergillus spp.* and their fusants showed cellulase enzyme activity with carboxymethyl cellulose as substrate ranging from 0.87 to 4.53 U/ml where Nid was the highest cellulase producer. The fungal utilization of low-cost sugarcane bagasse (SCB) based SSF media to produce lignocellulolytic enzymes minimizes the enzymatic production expenses [8, 22]. Also, SCB was the best inducer for exoglycanase, endoglucanase, and β-glucosidase production and was revealed to be the best carbon source, presumably because of its high cellulose and low lignin contents [23].

Fungal protoplasts are an important tool in physiological and genetic research [24, 25]. Hence, protoplast fusion is one of the crucial methods [26] and a powerful tool for promoting genetic recombination and creating superior hybrid strains [27–30]. Isolation, fusion, and regeneration of protoplasts have been achieved in the genus *Aspergillus* mainly in the strain improvement program to enhance enzyme production [31]. The contrasting character of the parental strains is required for selecting the fusants [9]. In that light and according to the morphological and growth rate differences between the *Aspergillus spp.*, we conducted protoplast fusion and the resultant fusants tested for cellulase activity as well as the parental strains to observe strain improvement. The goal of the current work was to isolate the protoplasts from *Aspergillus* species and perform interspecific protoplast fusion in order to look into the possibility of increasing the extracellular synthesis of carboxymethyl cellulase (CMCase) in the progenies of the fusant. The results indicated that protoplast fusion technique successfully generated hybrid strain (PFNT) with enhanced and rapid cellulase production capabilities (3.11 U/ml by the second Incb. day) compared to the parental strain Tam highest activity (2.368 U/ml by the fourth Incb. day) in the initial SSF experiment. Our results agreed with [32] where the native and heterologous enzymes of *A. nidulans* have been applied in numerous industrial processes. It is a versatile filamentous fungal cell factory that is a potential resource for industrial enzymes such as cellulases, β-glucosidases, and hemicellulases from low-cost substrates such as lignocellulosic waste. Also, *A. tamarii* was identified as the one with the highest xylanase activity of 9 strains from the genus *Aspergillus* [23]. On the other hand, the fusant PFOT showed an unremarkable increase and the fusant PFFT exhibited a decrease in activity as compared to the parents. These findings suggest that protoplast fusion may involve partial or total genetic recombination, which may have had unfavorable consequences for fusants which is consistent with [9].

Cellulase-secreting microorganisms belonging to different species or genera may have distinct cellulase genes and control systems. Protoplast fusion could be used to recombine them, and strong screening methods could then be applied to select the superior recombinant strains [33]. Therefore, in the presented study, we developed a useful strain as a single source of cellulase among other enzymes; it was intended to incorporate the characteristics of two parental species of *Aspergillus* belonging to the high cellulase-producing species *A. nidulans* and the high xylanase-producing species *A. tamarii*, by fusing their protoplasts. The resultant fusant PFNT showed enhanced cellulase activity compared with the high xylanase-producing parent (Tam) which means that protoplast fusion achieved a strain improvement and added a new feature to the *A. tamarii* as cellulase producer among its other well-known advantages in the enzymes production. Based on that, the fusant PFNT with enhanced cellulase activity was selected for further optimization to elevate the enzyme activity level.

It’s crucial to shorten the production time to maximize the efficiency of the enzyme production process and finish more batches in a shorter period. In addition, the ideal duration for achieving the intended level of enzyme production is contingent upon the medium’s initial inoculum moisture content, concentration, incubation temperature, and interaction of an organism with a substrate [34]. Oberoi et al. [35] reported that a fall in enzyme activity after 72 or 96 h, for different isolates could possibly be due to the organism going to a stationary phase of growth; the nutrients running out, the fermentation medium producing additional byproducts, or a combination of all of the aforementioned causes. The current study agreed with that where enzyme activity in the parental strain (Nid), and the fusant (PFNT) was higher after 2 days of incubation and decreased with 4th day, while the parental strain (Tam) took a longer time to enter the stationary phase of growth. Initially, fungi break down the easily accessible carbohydrates(sugars) to create hydrolytic enzymes, However, over time, as the concentration of sugar decreases, the fungus start using these hydrolytic enzymes to make sugars, which results in a decrease in enzyme activity [35].

To increase cellulase production by the protoplast fusant *Aspergillus* PFNT through the two stages (PB and BB), a sequential optimization technique was used. Normally, it is important to examine as many variables as possible and understand their significance when examining the factors influencing the development of specific secondary metabolites [36]. A helpful quick screening procedure is offered by the PB design, and it statistically calculates the relevance of numerous factors in a single trial, saving time and preserving strong evidence for each element. Even though this model does not take interaction into account, the screening program does not place a high premium on looking at how these numerous elements interact. Only the most beneficial and successful variables would be kept for further optimization, while those that significantly harmed the bioprocess may be excluded from all upcoming experiments [14]. In this investigation, PB results showed a wide range of cellulase activity from 1.4 up to 3.7 U/ml. This variant illustrates how critical medium-level optimization is to boost productivity. The study of the regression coefficients, *t-test*, and *p-value* for the seven variables clearly shows that SCB, pH, and incubation temperature were the three factors that had the greatest impact on the production of cellulase activity, with *p-values* of 0.009458, 0.055389, and 0.011698, respectively.

The goal of RSM use in this study was to increase cellulase yield by reducing time, energy, costs, and manufacturing errors. Since changing the independent factors caused the dependent elements to change automatically, this practice had proven quite successful [37]. SCB, pH and incubation temperature greatly impacted cellulase synthesis, according to RSM analysis. In agreement with [35, 38] where the amount and growth rate of an organism are both greatly influenced by temperature, which also has a significant impact on the manufacturing of the desired output. Whereas lower temperature inhibits the movement of substrate through the cells, lowering product yields. The thermal denaturation of the metabolic pathway’s enzymes causes less product synthesis at higher temperatures, which raises the maintenance energy demand for cellular growth. The results obtained in the present study showed that when three different temperatures 25, 30, and 35˚C were tested the higher activity attained with 31.8 ˚C as incubation temperature.

In line with our findings, which revealed that high enzymatic activity was detected at pH 4, Imran et al. [39] demonstrated that increased initial pH content for SSF had a negative or restricting influence on cellulase. Experimental validation and comparison with the model’s expected optimum resulted in the determination of the ideal circumstances obtained from the optimization plan. The polynomial model estimates a value of 7.689 U/ml, while the estimated cellulase activity was 8.72 U/ml. This high level of accuracy (113.4%) shows that the model has been verified. under ideal circumstances. Enzyme activity in the optimized medium was also approximately 2.8-fold higher than it was under the initial conditions. This illustrated the value of the optimization process and its necessity. Cellulase was found to dramatically increase phenolic extraction yields and antioxidant capabilities, according to [40], because it was able to hydrolyze the product’s cell walls, allowing the release and recovery of these compounds which is consistent with our findings. Along with the rise in cellulase load, Ghandahari et al. [41] also noted an increase in the extraction yield of phenolic compounds.

## Conclusion

The results indicate that protoplast fusion can be an effective strategy for improving cellulase production in *Aspergillus* species. The use of RSM allowed for the identification and optimization of critical process variables, leading to increased enzyme production. This research demonstrates the successful development of cellulase through protoplast fusion of *Aspergillus* species and its statistical optimization using RSM. The optimized conditions significantly enhanced cellulase production, providing a basis for further scale-up and application in various industries. Future studies should focus on downstream processing and purification techniques to maximize the commercial potential of the developed cellulase.

## Data Availability

No datasets were generated or analysed during the current study.

## Abbreviations

PFFT: Protoplast fusant from A. flavus X A. terreus
PFNT: Protoplast fusant from A. nidulans X A. tamari PFOT:Protoplast fusant from A. oryzae X A. tubingensis
PBD: Plackett-Burman design
BBD: Box Behnken design
CMCase: Carboxymethyl Cellulase
SCB: Sugarcane bagasse
WB: Wheat bran
Temp.: Temperature
PEG: polyethylene glycol
PDA: Potato Dextrose Agar
SSF: Solid-State Fermentation
CMC: Carboxymethyl Cellulose
RSM: Response surface methodology
DPPH: 1,1-diphenyl-2-picrylhydrazyl
RSA: Radical scavenging activity
Flv: Aspergillus flavus
Nid: Aspergillus nidulans
Ory: Aspergillus oryzae
Tam: Aspergillus tamarii
Ter: Aspergillus terreus
Tub: Aspergillus tubingensis
