# Correction: Enhancement of cellulolytic enzyme production from intrageneric protoplast fusion of *aspergillus* species and evaluating the hydrolysate scavenging activity

**DOI:** 10.1186/s12934-024-02377-2

**Published:** 2024-05-13

**Authors:** Doaa A. Goda, Huda M. Shakam, Mai E. Metwally, Hager A. Abdelrasoul, Mohamed M. Yacout

**Affiliations:** 1 Bioprocess Development Department, Genetic Engineering and Biotechnology Research Institute (GEBRI), City of Scientific Research and Technological Applications (SRTA-City), Universities and Research Institutes Zone, P.O. 21934, New Borg El-Arab City, Alexandria, Egypt; 2 Genetics Department, Faculty of Agriculture (El-Shatby), Alexandria, Egypt

**Correction to: Microbial Cell Factories**

10.1186/s12934-024-02343-y

Following publication of the original article [1], the authors identified an error in Mohamed M. Yacout’s affiliation .The affiliation was incorrectly given as “Bioprocess Development Department, Genetic Engineering and Biotechnology Research Institute (GEBRI), City of Scientific Research and Technological Applications (SRTA-City), Universities and Research Institutes Zone, P.O. 21,934, New Borg El-Arab City, Alexandria, Egypt”, but should have been “Genetics Department, Faculty of Agriculture (El-Shatby), Alexandria, Egypt ”.

This error is corrected in the affiliations list below and the original article [1] has been revised.
